# Inhibitory effects of the JAK inhibitor CP690,550 on human CD4^+ ^T lymphocyte cytokine production

**DOI:** 10.1186/1471-2172-12-51

**Published:** 2011-08-31

**Authors:** Kiyoshi Migita, Taiichiro Miyashita, Yasumori Izumi, Tomohiro Koga, Atsumasa Komori, Yumi Maeda, Yuka Jiuchi, Yoshihiro Aiba, Satoshi Yamasaki, Atsushi Kawakami, Minoru Nakamura, Hiromi Ishibashi

**Affiliations:** 1 Department of Rheumatology and Clinical Research Center, NHO Nagasaki Medical Center, Kubara 2-1001-1, Omura 856-8652, Japan; 2 Department of Rheumatology, Nagasaki University Hospital, Sakamoto 1-7-1 Nagasaki 852-8501, Japan

**Keywords:** CP690, 550, cytokines, Janus kinase, signal transducers and activators of transcription, T lymphocytes

## Abstract

**Background:**

The new JAK3 inhibitor, CP690,550, has shown efficacy in the treatment of rheumatoid arthritis. The present study was undertaken to assess the effects of CP690,550 on cytokine production and cellular signaling in human CD4^+ ^T cells.

**Results:**

CD4^+ ^T cells produced IL-2, IL-4, IL-17, IL-22 and IFN-γ in following stimulation with a CD3 antibody. At the optimal concentration, CP690,550 almost completely inhibited the production of IL-4, IL-17, IL-22 and IFN-γ from these activated CD4^+ ^T cells, but only had marginal effects on IL-2 production. Moreover CP690,550 inhibited anti-CD3-induced phosphorylation of STAT1, STAT3, STAT4, STAT5, and STAT6, but not the TCR-associated phosphorylation of ZAP-70.

**Conclusions:**

Therefore, CP690,550-mediated modification of the JAK/STAT pathway may be a new immunosuppressive strategy in the treatment of autoimmune diseases.

## Background

Janus kinases (JAKs) are cytoplasmic tyrosine kinases that participate in the signaling of cell surface receptors, particularly cytokine receptors [1]. Ligand-cytokine receptor binding induces activation of JAKs, which initiate signaling by phosphorylating cytokine receptors and creating docking sites for signaling proteins, known as signal transducers and activators of transcription (STATs) [2]. JAKs catalyze STAT phosphorylation to facilitate STAT dimerization, transport to the nucleus and ultimately regulate gene expression [3]. Of the members of the JAK family, JAK3 has features that make it a potentially attractive target for immunosuppression, since JAK3 associates with the common gamma (cγ) chain, which is shared by receptors of IL-2, IL-4, IL-7, IL-9, IL-15 and IL-21 [4]. Moreover, mice and humans with a heritable absence or mutation of JAK3 express a severe combined immunodeficiency phenotype [5,6]. Therefore selective inhibition of JAK3 represents an optimal strategy for immunosuppression and the treatment of autoimmune diseases [7]. CP690,550, a JAK3 inhibitor that is currently in clinical trials, has been shown to significantly reduce joint inflammation in rheumatoid arthritis (RA) [8,9]. The JAK/STAT pathways influence cell-fate decisions made by differentiating naïve T cells, helping to control their development into Th1 Th2 and Th17 cells [10]. Commitment to the Th1 lineage requires STAT1- and STAT4-dependent mechanisms that induce IFN-γ and T-bet expression [11]. On the other hand, differentiation towards the Th2 developmental pathway requires STAT6 [12]. STAT3 has emerged as an important determinant of T cell differentiation towards the inflammatory Th17 T cell lineage [13]. As the JAK/STAT pathway plays a pivotal role in T cell differentiation and cytokine signaling in T cells, we postulated that selective inhibition of JAKs with CP690,550 would modulate T cell functions and characteristics. In this study, we assessed the effects of a pharmacological inhibitor of JAK3, CP690,550, on gene expression and secretion of cytokines by human CD4^+ ^T cells. We also examined whether CP690,550 affected the STAT activation status in activated CD4^+ ^T cells.

## Results

### Cytokine production by CD4 T cells is greatly reduced by CP690,550

**Figure 1:**
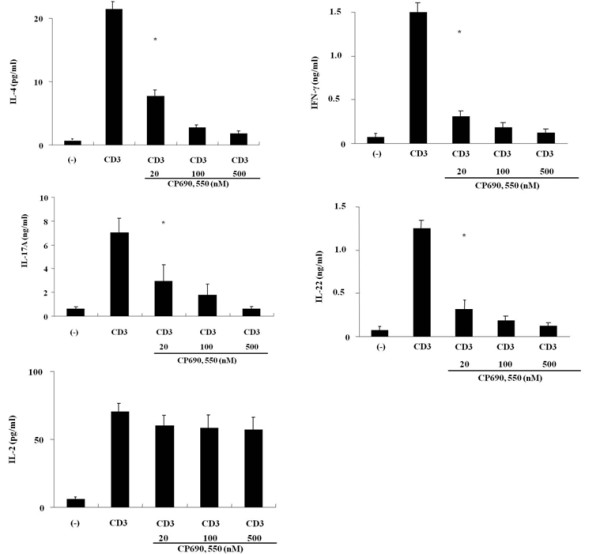
**Effects of CP690, 550 activated CD4^+ ^T cell cytokine production**. CD4^+ ^T cells were stimulated with CD3 monoclonal antibodies in the presence or absence of CP690,550 for 48 hr. Supernatants were collected and the levels of IL-2, IL-4 (A), IFN-γ (B), IL-17A (C), IL-22 (D) and IL-2 (E) were measured by ELISA. The figure shows the means ± SD of the three independent experiments performed in triplicate. * *p *< 0.001 vs CD3 Ab-stimulated lymphocytes.

To investigate the potential role of JAKs in T cell activation, CD4^+ ^T cells isolated from healthy subjects were stimulated with a CD3 monoclonal antibody in the presence of CP690,550 for 2 days. As shown in Figure 1, freshly isolated CD4^+ ^T cells secreted a significant amount of IL-4 (A), IFN-γ (B), IL-17A (C) and IL-22 (D) in response to stimulation with the CD3 antibody. CP690,550 completely abrogated secretion of these cytokines from CD4^+ ^T cells. Meanwhile, CP690,550 did not affect the secretion of IL-2 by anti-CD3-stimulated CD4^+ ^T cells (Figure 1E).

**Figure 2:**
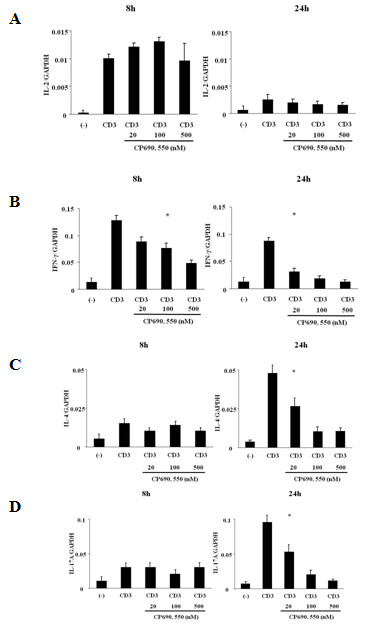
**Effects of CP690, 550 on cytokine genes expression in activated CD4^+ ^T cells**. CD4^+ ^T cells were stimulated with CD3 monoclonal antibodies in the presence or absence of CP690,550. After 8-hr or 24-hr stimulation, total RNA was extracted and IL-2 (A), IFN-γ (B), IL-4 (C) and IL-17A (D) mRNA expression was measured by real-time PCR. * *p *< 0.001 vs CD3 Ab-stimulated lymphocytes. The results show a representative result from three independent experiments.

**Figure 3:**
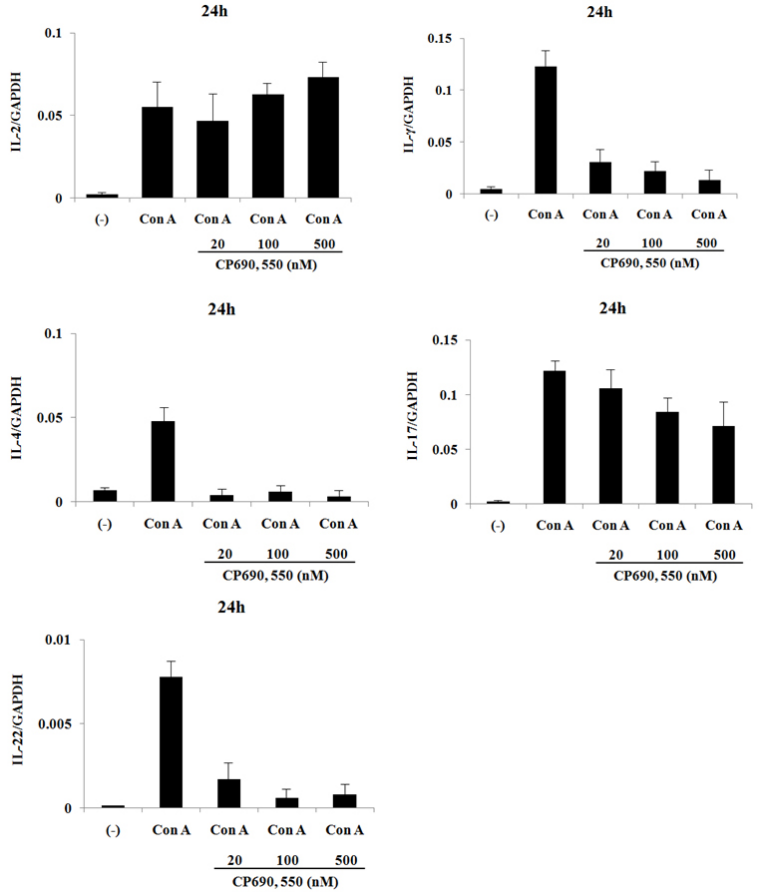
**Effects of CP690, 550 on cytokine genes expression in Con-A activated CD4^+ ^T cells**. CD4^+ ^T cells were stimulated with concanavalin A in the presence or absence of CP690,550. After 24-hr stimulation, total RNA was extracted and IL-2, IFN-γ, IL-4, IL-17A and IL-22 mRNA expression was measured by real-time PCR. The results show a representative result from three independent experiments.

**Figure 4:**
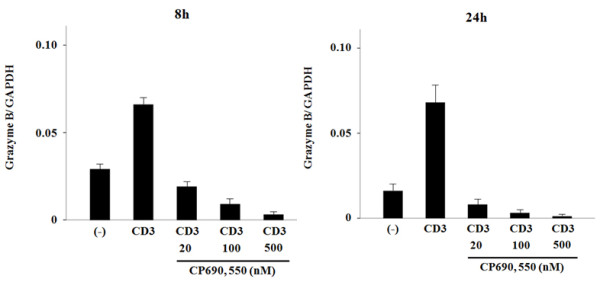
**Effects of CP690, 550 on granzyme B mRNA expression in activated CD8^+ ^T cells**. CD8^+ ^T cells were stimulated with CD3 monoclonal antibodies in the presence or absence of CP690,550. After 8-hr or 24-hr stimulation, total RNA was extracted and granzyme B mRNA expression was measured by real-time PCR. The results show a representative result from two independent experiments.

To confirm these findings, we examined mRNA levels of these cytokines in CD4^+ ^T cells using real-time PCR. Stimulation for 8 hrs with the CD3 antibody induced IL-2 (Figure 2A) and IFN-γ mRNA (Figure 2B) expression in CD4^+ ^T cells. The increased IFN-γ mRNA levels were down regulated by CP690,550 (Figure 2B), whereas the anti-CD3-stimulated expression of IL-2 mRNA was not affected (Figure 2A). The expression of IL-4 and IL-17 mRNA was marginally induced after 8 hrs of anti-CD3 stimulation. In contrast, when CD4^+ ^T cells were stimulated with the CD3 antibody for 24 hrs, expression of IL-4 and IL-17 mRNA was greatly induced (Figure 2C, D). Consistent with the protein data, the induction of IL-4 and IL-17 mRNA in anti-CD3-stimulated CD4^+ ^T cells was down regulated by CP690,550 (Figure 2C, D). We next examined the effects of CP690,550 on concanavalin A (Con A)-activated CD4^+ ^T cells. As shown in Figure 3, CP690,550 inhibited cytokine mRNA expression of Con A-activated CD4^+ ^T cells. Furthermore, CP690,550 inhibited granzyme B mRNA expressions in anti-CD3-stimulated CD8^+ ^T cells (Figure 4).

### Effects of CP690,550 on CD4^+ ^T cell proliferation

**Figure 5:**
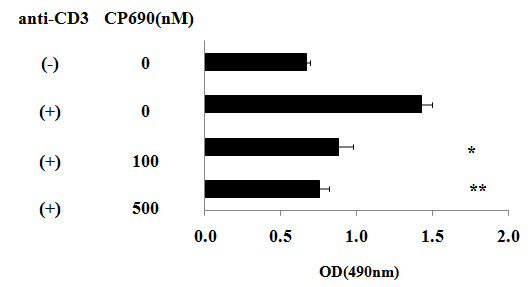
**Anti-proliferative effects of CP690,550 on CD3 Ab activated CD4^+ ^T cells**. CD4^+ ^T cells were stimulated with CD3 monoclonal antibodies in the presence or absence of CP690,550. After 48-hr, proliferation was determined by the XTT assay. * *p *= 0.023 vs CD3 Ab-stimulated lymphocytes. ** *p *= 0.010 vs CD3 Ab-stimulated lymphocytes. The results are expressed as the mean ± SD of two independent experiments.

**Figure 6:**
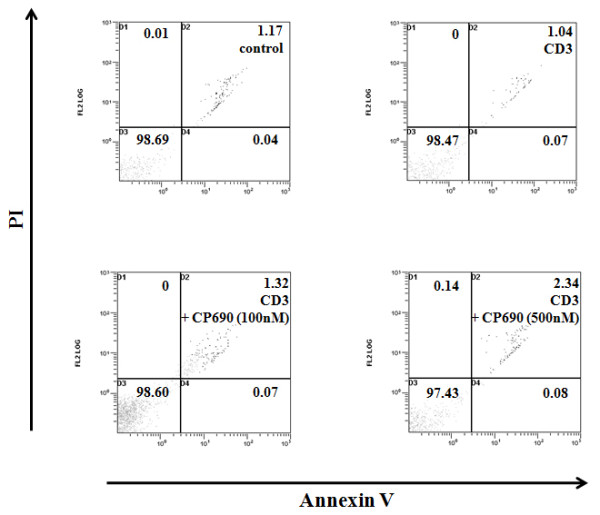
**Effects of CP690,550 on the apoptosis induction in CD3 Ab activated CD4^+ ^T cells**. CD4^+ ^T cells were stimulated with CD3 monoclonal antibodies in the presence or absence of CP690,550. After 24-hr, the cells were stained with FITC-conjugated Annexin V and PI and were then analyzed by flow cytometry. Ten thousand cells were measured and plotted. The proportion of cells residing in each quadrant is expressed as a percentage. Results are representative of 3 independent results.

Next, we examined the effects of CP690,550 on T cell proliferation. As shown in Figure 5, CP690,550 pre-treatment significantly inhibited anti-CD3-induced CD4^+ ^T cell proliferation. We also investigated whether CP690,550 pre-treatment induces apoptosis in anti-CD3-activated CD4^+ ^T cells. CP690,550 pre-treatment did not result in an increase in the percentages of annexin V-positive/PI-negative early apoptotic cells (Figure 6).

### CP690,550 abrogated CD3-induced phosphorylation of STATs in CD4^+ ^T cells

**Figure 7:**
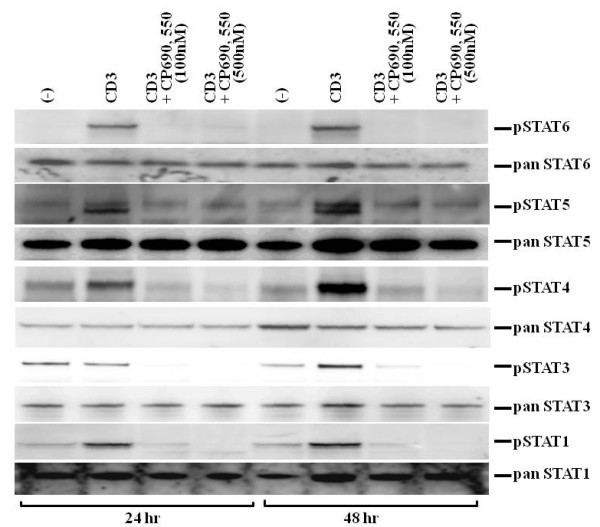
**CP690,550 inhibited STATs phosphorylation in CD3 Ab-activated CD4^+ ^T cells**. CD4^+ ^T cells were stimulated with CD3 monoclonal antibodies in the presence or absence of CP690, 550 for 24 hr and 48 hr. STAT1, STAT3, STAT4, STAT5 and STAT6 phosphorylations were measured by western blotting. The results show a representative blot from three independent experiments.

The STAT transcription factors help mediate the differentiation of CD4^+ ^T cells into each of the T helper (Th) subsets and are involved in lineage-specific expression of cytokines. To determine whether CP690,550 affected the phosphorylation of the STATs in the T cell activation process, we assessed the phosphorylation-status of STATs in anti-CD3-stimulated T cells (Figure 7). After 24 hr of anti-CD3 stimulation, phosphorylation of STAT1, STAT4, STAT5 and STAT6 was induced in CD4^+ ^T cells. These phosphorylation events were inhibited by 100 nM CP690,550 at 24 and also at 48 hrs. CD3 antibody stimulation did not augment the phosphorylation of STAT3 after 24 hrs, but this was evident after 48 hrs. Similarly, CP690,550 also inhibited the late phase STAT3 phosphorylation in activated CD4^+ ^T cells.

### CP690,550 did not affect CD3-induced phosphorylation of ZAP-70 in CD4^+ ^T cells

**Figure 8:**
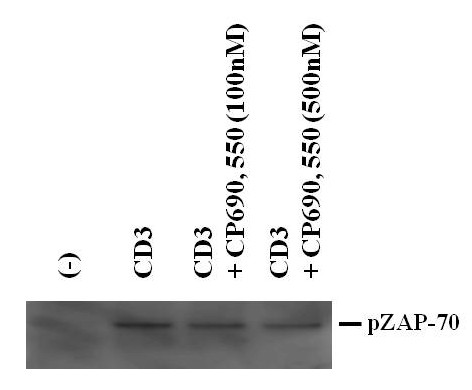
**CP690,550 did not affect ZAP-70 phosphorylation in CD3 Ab-activated CD4^+ ^T cells**. CD4^+ ^T cells were stimulated with CD3 monoclonal antibodies in the presence or absence of CP690,550 for 30 min. ZAP-70 phosphorylation was analyzed by western blotting. The results show a representative blot from three independent experiments.

Finally, we tested the effect of CP690,550 on TCR-directed signaling by assessing TCR-associated phosphorylation of ZAP-70. ZAP-70 phosphorylation was induced in CD4^+ ^T cells after stimulation for 30 min with a CD3-antibody, but CP690,550 exhibited no inhibitory effect on ZAP-70 phosphorylation (Figure 8).

## Discussion

It is well-established that cytokines play a critical role in regulating autoimmunity and inflammation, since targeting cytokines has been shown to be effective in the treatment of RA [14]. However, targeting intracellular cytokine signaling using protein kinase inhibitors, appears to be a new strategy for immunosuppression [15]. CP690,550, a JAK inhibitor that is currently in clinical trials, has shown significant efficacy in the treatment of RA [8,9]. However, the precise mechanisms responsible for CP690,550-mediated immunosuppression have not been elucidated.

Naïve CD4^+ ^T cells can differentiate into two major distinct phenotypes, Th1 and Th2 cells, which are characterized by polarized cytokine expression patterns [16]. TCR signaling plays an important role in this differentiation by inducing Th-specific cytokines [17]. Naïve CD4^+ ^T cells are capable of producing IFN-γ and IL-4 when they are stimulated with peptide/MHC class II complexes on antigen-presenting cells (APC) [18]. The endogenous production of IFN-γ has been shown to be sufficient for Th1 differentiation, whereas IL-4 is critical for Th2 differentiation [19]. In this study, we examined the effects of CP690,550 on the production of these cytokines from CD4^+ ^T cells activated by CD3-stimulation. Our data indicated that CP690,550, a JAK3 inhibitor, abrogated IFN-γ and IL-4 production from CD4^+ ^T cells trigged a CD3 antibody.

JAKs are cytoplasmic tyrosine kinases that participate in the signaling of the cytoplasmic receptor of the cγ chain family receptors for IL-2, IL-4, IL-7, IL-9, IL-15 and IL-21 [20]. Ligand receptor-induced activation of JAKs initiates signaling by phosphorylation of the cytokine receptors and catalyzes STAT phosphorylation, facilitating STAT dimerization, transport to the nucleus and gene regulation [3]. Therefore, it is possible that CP690,550 affects the STAT activation processes. Th1 differentiation depends on signaling through the IFN-γ receptor, the IL-12 receptor and their downstream transcription factors, STAT1 and STAT4 [21]. Similarly, mature Th2 differentiation depends on the IL-4 receptor and its downstream transcription factor STAT6 [12]. We demonstrated that CP690,550 inhibited STAT1, STAT4 and STAT6 activation, in addition to IFN-γ and IL-4 production, in TCR-stimulated CD4^+ ^T cells. These findings suggest that CP690,550 inhibits the production of Th1- or Th2-specific cytokines, as well as cytokine receptor signaling.

The JAK1-STAT1 and JAK2-STAT4 signaling pathways are known to be important for Th1 cell differentiation, but JAK3 has not previously been implicated in Th1 cell differentiation [22]. In contrast, JAK3 is essential for Th2 differentiation, since it plays an essential role in IL-4 signaling [23]. Our data suggest that CP690,550, a JAK3 inhibitor, affected the CD3-induced Th1-related activation of JAK1-STAT1 or JAK2-STAT4 in addition to the Th2-related activation of JAK3-STAT6. CP690,550 was originally believed to be a JAK3 inhibitor [24], but it has now become clear that this compound also inhibits JAK1 and JAK2 with similar IC_50_-values [25,26]. Therefore, it is possible that CP690,550 not only affects the induction of Th2-related cytokines, but also the induction of Th1-related cytokines via JAK1 and JAK2, in addition to JAK3.

More recently, the IL-2-JAK3-STAT5 axis has been demonstrated to regulate Th1 cell differentiation, suggesting that IL-2-mediated JAK3-STAT5 signaling may generically operate in the production of Th1-related cytokines [27]. We also demonstrated that the CD3 antibody-induced phosphorylation of STAT5 was down regulated by CP690,550, suggesting that JAK3 inhibition by CP690,550 down regulated STAT5-dependent cytokine signaling.

It is particularly interesting to evaluate the contribution of Th17 cells to the pathogenesis of RA, as IL-17 is involved in immune-mediated articular damage [28]. The current view of human Th17 development is that IL-6 and IL-23 are key cytokines that initiate differentiation and maintenance of this lineage [29]. Th17 cells are also major producers of IL-22, in addition to IL-17 [30]. In contrast with mouse T cells, TCR stimulation alone has been shown to be sufficient to induce both IL-17 in humans T cells [31]. Indeed, anti-CD3-stimulation induced secretion of both IL-17 and IL-22 from CD4^+ ^T cells in our study. Our data also demonstrated that CP690,550 almost completely inhibited CD3-induced production of IL-17 or IL-22 from human CD4^+ ^T cells. Recent evidence has supported a new role for STAT3, in the differentiation of Th17 cells [32]; STAT3-deficient mice fail to produce IL-17 and restoration of STAT3 rescues the IL-17 defect [33]. It has also been reported that activated STAT3 binds directly to the STAT3-binding sites in the promoter of the IL-17 gene, increasing its expression [34]. These findings indicate that STAT3 signaling is important for IL-17 induction and Th17 differentiation. In this study, anti-CD3 stimulation activated STAT3 during the late phase of T cell activation (i.e., 48 hrs), which was blocked by CP690,550. This impaired STAT3 activation may have partially contributed to the suppression of Th17-related cytokine production by CP690,550.

Resting T cells require the sequential activation of two signaling pathways to differentiate. First, antigen stimulation of the TCR/CD3 complex leads to the expression of IL-2 and IL-2R. Second, the binding of IL-2 to its cognate receptor leads to JAK/STAT activation [35]. In our study, CP690,550 did not directly affect TCR-mediated signaling, i.e., ZAP-70 activation. However, CP690,550 abrogated the subsequent activation of the STATs (STAT1, 3, 4, 5, 6). These findings suggest that CP690,550 induces immunosuppression by affecting cytokine receptor-associated JAK/STAT signaling, which is downstream of the TCR-signaling pathway. These findings may provide important information concerning the mechanisms of CP690,550-mediated immunosuppression in the treatment of RA.

In T cell activation, the first signal is triggered by the engagement of the TCR with the antigen after presentation by the antigen-presenting cell (APC), which is then ultimately transmitted by the final signal triggered by cytokine engagement of their receptors on T cells via the JAK/STAT pathway [36]. In this study, we demonstrated that CP690,550 abrogated the TCR-triggered production of cytokines by affecting the JAK/STAT signaling pathway, not the TCR-mediated signaling pathway. We believe that small molecule inhibitors that target JAKs may provide alternative therapeutic strategies for immunosuppression in contrast to classical immunosuppressants, such as calcineurin inhibitors, that target TCR-mediated signaling.

## Conclusion

CP690, 550 inhibited anti-CD3-induced IFN-γ, IL-4 and IL-17 production from CD4^+ ^T cells without affecting IL-2 production. CP690, 550 also abrogated anti-CD3-induced STAT1, STAT3, STAT4, STAT5 and STAT6 activation in CD4^+ ^T cells. Modification of the JAK/STAT pathway using small molecule inhibitors may provide a new strategy for immunosuppression.

## Methods

### Reagents

JAK inhibitor, CP690,550 was obtained from Axon Biochemicals (Postbus, Netherlands). Human CD3 monoclonal antibody (UCTH1) was purchased from BD Biosciences (Franklin Lakes, NJ, USA). Phospho-specific and pan antibodies against STAT-1 (Tyr701), STAT-3 (Tyr705), STAT-4 (Tyr639), STAT-5 (Tyr694), STAT-6 (Tyr641) and phospho-specific ZAP-70 (Tyr493) were purchased from Cell Signaling Technology (Beverly, MA USA).

### Isolation of CD4^+ ^and CD8^+ ^T lymphocytes

Peripheral blood mononuclear cells were isolated from peripheral blood of healthy volunteers by Ficoll-Hypaque isopycnic centrifugation. Informed consent was obtained from each of the individuals. Next, CD4^+ ^or CD8^+ ^T lymphocytes were positively selected by use of magnetic CD4 or CD8 MicroBeads (Miltenyi Biotec, Bergisch Gladbach, Germany), as described by the manufacturer. More than 98% of the isolated cells were positive for CD3 and CD4, as measured by flow cytometry. T cells were cultured in standard 24-well flat-bottom microtiter plates (BD Biosciences; Falcon 3047) at 1.0 × 10^6 ^cells/ml in RPMI 1640 supplemented with 10% FBS at 37°C in a humidified incubator with 5% CO_2_.

### Cytokine enzyme-linked immunosorbent assay (ELISA)

The amounts of IFN-γ, IL-2, IL-4, IL-17A and IL-22 in the supernatants of the cultured CD4^+ ^T lymphocytes was measured using commercial ELISA kits (R&D Systems, Minneapolis, MN) according to the manufacturer's instructions

### Real-time polymerase chain reaction (PCR) analysis

Total cellular RNA was extracted with Trizol (Invitrogen, Carlsbad, CA) according to the manufacturer's protocol. First-strand cDNA was synthesized from 1 μg of total cellular RNA using an RNA PCR kit (Takara Bio Inc., Otsu, Japan) with random primers. Amplification of the IFN-γ, IL-4, IL-2, IL-17A and granzyme B was performed on a Light Cycler (Roche Diagnostics, Mannheim, Germany) using specific primers. The housekeeping gene fragment of glyceraldehydes-3-phosphates dehydrogenase (GAPDH) was used for verification of equal loading.

### Western blotting

Cultured CD4^+ ^T lymphocytes were washed with ice-cold PBS and lysed with a lysis buffer (1% Nonidet P 40, 50 mM Tris, pH 7.5, 100 mM NaCl, 50 mM NaF, 5 mM EDTA, 20 mM β-glycerophosphate, 1.0 mM sodium orthovanadate, 10 μg/mL aprotinin and 10 μg/mL leupeptin) for 20 minutes at 4°C. Insoluble material was removed by centrifugation at 15,000 × g for 15 min at 4°C. The supernatant was saved and the protein concentration was determined using the Bio-Rad protein assay kit (Bio Rad, Hercules, CA). An identical amount of protein (50 μg) for each lysate was subjected to 10% SDS-polyacrylamide gel electrophoresis, and then transferred to a nitrocellulose membrane. Western blot analysis using phospho-specific STAT and anti-pan STAT antibodies was performed with an ECL Western blotting kit (Amersham, Little Chalfont, UK).

### XTT cell proliferation assay

CD4^+ ^T cell proliferation was assessed by the 2, 3-bis- [2-methoxy-4-nitro-5-sulfophenyl]-2H-tetrazolium-5-carboxanilide (XTT) assay (Roche, Mannheim, Germany). In briefly, CD4^+ ^T cells (10^4 ^cells/well) were plated into 96-well plates in the presence or absence of various concentrations of CP690, 550. After stimulation with a CD3 antibody for 48 hr, 50 μl of XTT labeling mixture was added to each well. Finally, the optical density was measured at 490 nm with a reference wave length at 690 nm in a microplate reader (Thermo Scientific, Tokyo, Japan).

### Apoptosis detection assay

Apoptosis and cell death were measured with a MEBCYTO Apoptosis Kit (MBL, Nagoya). In brief, after stimulation with a CD3 antibody, cells were stained with 40 μg/ml propidium iodide (PI) and annexin V-FITC. The percentage of annexin V-positive/PI-negative early apoptotic cells and PI-positive dead cells were determined using an Epics XL flow cytometer (Beckman Coulter).

### Statistical analysis

Differences between groups were examined for statistical significance using the Wilcoxon-Mann-Whitney U test. *P *values less than 0.05 were considered statistically significant.

## Competing interests

The authors declare that they have no competing interests.

## Authors' contributions

KM, AK, YA, YM and YJ carried out cell culture and biochemical analysis. TK, YI, TM and MN participated in the design of the study and performed the statistical analysis. SY, AK and HI conceived the study, participated in its design and coordinated and helped to draft the manuscript. All authors read and approved the final manuscript.

## References

[B1] Schindler C Levy DE Decker T JAK-STAT signaling: from interferons to cytokines J Biol Chem 2007 282 20059 63 10.1074/jbc.R700016200 17502367

[B2] Leonard WJ O'Shea JJ Jaks and STATs: biological implications Annu Rev Immunol 1998 16 293 322 10.1146/annurev.immunol.16.1.293 9597132

[B3] Levy DE Darnell JE Jr Stats: transcriptional control and biological impact Nat Rev Mol Cell Biol 2002 3 651 62 12209125 10.1038/nrm909

[B4] O'Shea JJ Pesu M Borie DC Changelian PS A new modality for immunosuppression: targeting the JAK/STAT pathway Nat Rev Drug Discov 2004 3 555 64 10.1038/nrd1441 15232577

[B5] Noguchi M Yi H Rosenblatt HM Filipovich AH Adelstein S Modi WS McBride OW Leonard WJ Interleukin-2 receptor gamma chain mutation results in X-linked severe combined immunodeficiency in humans Cell 1993 73 147 57 10.1016/0092-8674(93)90167-O 8462096

[B6] Nosaka T van Deursen JM Tripp RA Thierfelder WE Witthuhn BA McMickle AP Doherty PC Grosveld GC Ihle JN Defective lymphoid development in mice lacking Jak3 Science 1995 270 800 2 10.1126/science.270.5237.800 7481769

[B7] Pesu M Laurence A Kishore N Zwillich SH Chan G O'Shea JJ Therapeutic targeting of Janus kinases Immunol Rev 2008 223 132 42 10.1111/j.1600-065X.2008.00644.x 18613833 PMC2634846

[B8] Kremer JM Bloom BJ Breedveld FC Coombs JH Fletcher MP Gruben D Krishnaswami S Burgos-Vargas R Wilkinson B Zerbini CA Zwillich SH The safety and efficacy of a JAK inhibitor in patients with active rheumatoid arthritis: Results of a double-blind, placebo-controlled phase IIa trial of three dosage levels of CP-690,550 versus placebo Arthritis Rheum 2009 60 1895 905 10.1002/art.24567 19565475

[B9] Coombs JH Bloom BJ Breedveld FC Fletcher MP Gruben D Kremer JM Burgos-Vargas R Wilkinson B Zerbini CA Zwillich SH Improved pain, physical functioning and health status in patients with rheumatoid arthritis treated with CP-690,550, an orally active Janus kinase (JAK) inhibitor: results from a randomised, double-blind, placebo-controlled trial Ann Rheum Dis 2010 69 413 6 10.1136/ard.2009.108159 19587388

[B10] Murphy KM Reiner SL The lineage decisions of helper T cells Nat Rev Immunol 2002 2 933 44 10.1038/nri954 12461566

[B11] Szabo SJ Sullivan BM Peng SL Glimcher LH Molecular mechanisms regulating Th1 immune responses Annu Rev Immunol 2003 21 713 58 10.1146/annurev.immunol.21.120601.140942 12500979

[B12] Paul WE What determines Th2 differentiation, in vitro and in vivo? Immunol Cell Biol 2010 88 236 9 10.1038/icb.2010.2 20157328

[B13] Chen Z Laurence A O'Shea JJ Signal transduction pathways and transcriptional regulation in the control of Th17 differentiation Semin Immunol 2007 19 400 8 10.1016/j.smim.2007.10.015 18166487 PMC2323678

[B14] Scheinecker C Redlich K Smolen JS Cytokines as therapeutic targets: advances and limitations Immunity 2008 28 440 4 10.1016/j.immuni.2008.03.005 18400186

[B15] Cohen S Fleischmann R Kinase inhibitors: a new approach to rheumatoid arthritis treatment Curr Opin Rheumatol 2010 22 330 5 10.1097/BOR.0b013e3283378e6f 20164774

[B16] Murphy KM Reiner SL The lineage decisions of helper T cells Nat Rev Immunol 2002 2 933 44 10.1038/nri954 12461566

[B17] Zhu J Yamane H Paul WE Differentiation of effector CD4 T cell populations Annu Rev Immunol 2010 28 445 89 10.1146/annurev-immunol-030409-101212 20192806 PMC3502616

[B18] Seder RA Paul WE Acquisition of lymphokine-producing phenotype by CD4+ T cells Annu Rev Immunol 1994 12 635 73 10.1146/annurev.iy.12.040194.003223 7912089

[B19] Zhou L Chong MM Littman DR Plasticity of CD4+ T cell lineage differentiation Immunity 2009 30 646 55 10.1016/j.immuni.2009.05.001 19464987

[B20] Leonard WJ X-linked severe combined immunodeficiency: from molecular cause to gene therapy within seven years Mol Med Today 2000 6 403 7 10.1016/S1357-4310(00)01782-2 11006530

[B21] Murphy KM Ouyang W Szabo SJ Jacobson NG Guler ML Gorham JD Gubler U Murphy TL T helper differentiation proceeds through Stat1-dependent, Stat4-dependent and Stat4-independent phases Curr Top Microbiol Immunol 1999 238 13 26 10087648 10.1007/978-3-662-09709-0_2

[B22] Imada K Leonard WJ The Jak-STAT pathway Mol Immunol 2000 37 1 11 10.1016/S0161-5890(00)00018-3 10781830

[B23] Cote-Sierra J Foucras G Guo L Chiodetti L Young HA Hu-Li J Zhu J Paul WE Interleukin 2 plays a central role in Th2 differentiation Proc Natl Acad Sci USA 2004 101 3880 5 10.1073/pnas.0400339101 15004274 PMC374338

[B24] Changelian PS Flanagan ME Ball DJ Kent CR Magnuson KS Martin WH Rizzuti BJ Sawyer PS Perry BD Brissette WH McCurdy SP Kudlacz EM Conklyn MJ Elliott EA Koslov ER Fisher MB Strelevitz TJ Yoon K Whipple DA Sun J Munchhof MJ Doty JL Casavant JM Blumenkopf TA Hines M Brown MF Lillie BM Subramanyam C Shang-Poa C Milici AJ Prevention of organ allograft rejection by a specific Janus kinase 3 inhibitor Science 2003 302 875 8 10.1126/science.1087061 14593182

[B25] Williams NK Bamert RS Patel O Wang C Walden PM Wilks AF Fantino E Rossjohn J Lucet IS Dissecting specificity in the Janus kinases: the structures of JAK-specific inhibitors complexed to the JAK1 and JAK2 protein tyrosine kinase domains J Mol Biol 2009 387 219 32 10.1016/j.jmb.2009.01.041 19361440

[B26] Jiang JK Ghoreschi K Deflorian F Chen Z Perreira M Pesu M Smith J Nguyen DT Liu EH Leister W Costanzi S O'Shea JJ Thomas CJ Examining the chirality, conformation and selective kinase inhibition of 3-((3R,4R)-4-methyl-3-(methyl(7H-pyrrolo[2,3-d]pyrimidin-4-yl)amino)piperidin-1-yl)-3-oxopropanenitrile (CP-690,550) J Med Chem 2008 51 8012 8 10.1021/jm801142b 19053756 PMC2660606

[B27] Shi M Lin TH Appell KC Berg LJ Janus-kinase-3-dependent signals induce chromatin remodeling at the Ifng locus during T helper 1 cell differentiation Immunity 2008 28 763 73 10.1016/j.immuni.2008.04.016 18549798 PMC2587400

[B28] Park H Li Z Yang XO Nurieva R Wang YH Wang Y Hood L Zhu Z Tian Q Dong C A distinct lineage of CD4 T cells regulates tissue inflammation by producing interleukin 17 Nat Immunol 2005 6 1133 41 10.1038/ni1261 16200068 PMC1618871

[B29] Annunziato F Cosmi L Liotta F Maggi E Romagnani S Type 17 T helper cells-origins, features and possible roles in rheumatic disease Nat Rev Rheumatol 2009 5 325 31 10.1038/nrrheum.2009.80 19434074

[B30] Aujla SJ Kolls JK IL-22: a critical mediator in mucosal host defense J Mol Med 2009 87 451 4 10.1007/s00109-009-0448-1 19219418

[B31] Liu XK Lin X Gaffen SL Crucial role for nuclear factor of activated T cells in T cell receptor-mediated regulation of human interleukin-17 J Biol Chem 2004 279 52762 71 10.1074/jbc.M405764200 15459204

[B32] Korn T Bettelli E Oukka M Kuchroo VK IL-17 and Th17 Cells Annu Rev Immunol 2009 27 485 517 10.1146/annurev.immunol.021908.132710 19132915

[B33] Liu X Lee YS Yu CR Egwuagu CE Loss of STAT3 in CD4+ T cells prevents development of experimental autoimmune diseases J Immunol 2008 180 6070 6 18424728 10.4049/jimmunol.180.9.6070 PMC2435016

[B34] Durant L Watford WT Ramos HL Laurence A Vahedi G Wei L Takahashi H Sun HW Kanno Y Powrie F O'Shea JJ Diverse targets of the transcription factor STAT3 contribute to T cell pathogenicity and homeostasis Immunity 2010 32 605 15 10.1016/j.immuni.2010.05.003 20493732 PMC3148263

[B35] Berridge MJ Lymphocyte activation in health and disease Crit Rev Immunol 1997 17 155 78 9094451 10.1615/critrevimmunol.v17.i2.30

[B36] Ghoreschi K Laurence A O'Shea JJ Janus kinases in immune cell signaling Immunol Rev 2009 228 273 87 10.1111/j.1600-065X.2008.00754.x 19290934 PMC2782696

